# Gray Matters in Multiple Sclerosis: Cognitive Impairment and Structural MRI

**DOI:** 10.1155/2014/609694

**Published:** 2014-01-22

**Authors:** Silvia Messina, Francesco Patti

**Affiliations:** Department G.F. Ingrassia, Section of Neurosciences, University of Catania, Via S. Sofia 78, 95123 Catania, Italy

## Abstract

Multiple sclerosis (MS) is an immune-mediated disease affecting central nervous system (CNS). Although MS is classically considered a white matter (WM) disease, the involvement of gray matter (GM) in the pathogenic process has been confirmed by pathology studies and MRI studies. Impairment of cognitive domains such as memory, mental processing speed, attention, and executive function can occur from the early stage of the disease and tends to worsen over time, despite stable physical symptoms. WM demyelination is moderately correlated with CI, suggesting that probably WM abnormalities alone cannot fully explain the extent of clinical symptoms in MS, including CI. Several MRI techniques have shown the involvement of GM in MS and the association between GM damage, physical disability, and CI. The aim of this review is to provide an overview of CI and GM damage assessed by structural brain MRI.

## 1. Introduction

Multiple sclerosis (MS) is an immune-mediated disease affecting central nervous system (CNS), characterized by demyelination and axonal degeneration [1]. The etiology of MS is still unknown, although it is believed a complex interplay between genetic and environmental factors may have a role [2]. Although MS is classically considered a white matter (WM) disease, the involvement of gray matter (GM) in the pathogenic process has been confirmed by pathology studies [3, 4]. In the last years cognitive impairment (CI) has been recognized as an important feature of MS, affecting up to 65% of patients [5]. Impairment of cognitive domains such as memory, mental processing speed, attention, and executive function can occur from the early stage of the disease and tends to worsen over time, resulting in significant functional impairment at work and at home, despite minimal physical disability [6, 7]. It has been shown that WM demyelination is moderately correlated with CI, suggesting that probably WM abnormalities alone cannot fully explain the extent of clinical symptoms in MS, including CI [8, 9]. The definition of the pathogenic mechanism underlying the development of CI is crucial in order to identify novel biomarkers to monitor disease progression and therapy neuroprotective effect. Several MRI techniques have shown the involvement of GM in MS and the association between GM damage, physical disability, and CI [10–12]. The aim of this review is to provide an overview of CI and GM damage assessed by structural MRI. A review of functional imaging and its correlation with cognitive performance has not been included in the present paper.

## 2. Cognitive Impairment and Multiple Sclerosis

CI may affect patients at any stage of the disease, including patients with clinically isolated syndrome (CIS) [13]. A rarely occurring variant of MS, with an exclusive impairment of cognitive function, has also been described [14]. Cognitive deficit can be detected in benign MS patients (BMS) [15]; however it has been shown that CI tends to increase with worsening disability [7] and disease duration [16, 17] and could be associated with the onset of a progressive disease course [5, 17, 18]. Learning, memory, attention, processing speed, visuospatial abilities, and executive functions are affected most frequently in MS, whereas dementia and language deficits are less common [18, 19]. Large studies have shown that information processing speed and episodic memory are the more often impaired domains in MS patients [20]. Large longitudinal studies on cognition in MS often failed to show a significant cognitive decline, because of heterogeneity in the disease course and the potential influence of disease subtype and duration on CI [6]. According to these assumptions, it has been shown that only during the course of a sufficiently long follow-up period, cognitive dysfunction can emerge and progress with different degrees of severity [16]. The most sensitive tool to detect CI is neuropsychological testing (NPS). NPS testing, often perceived as time-consuming and expensive, should be administered by trained specialists and requires expertise, because physical disability (such as visual and other sensory deficits) can influence performance on cognitive test [21]. NPS testing, widely used in other diseases, such as mini mental state examination (MMSE), is not sensitive to detect CI in MS patients and was not adequately validated in MS population [6]. Two batteries of cognitive testing have been developed in the last years and have been widely used in clinical and research settings, the Rao's brief repeatable battery of neuropsychological test (BRB-N) [22] and the minimal assessment of cognitive function in MS (MACFIMS) [21]. Both batteries assess cognitive impairment covering the domain most commonly affected in MS, such as processing speed and working memory, new learning and episodic memory, and language. MACFIMS has the advantage to include the assessment of spatial processing speed and higher executive functions and to have stronger psychometric properties. BRB-N is less time-consuming and has been translated in several European languages [23, 24]. It has been demonstrated that MS patients tend to fail more often tests of information processing speed. There are two tests investigating processing speed in MS: symbol digit modality test (SDMT) and paced auditory serial addition task (PASAT). SDMT has been proposed as an alternative to PASAT in MS functional composite (MSFC), showing a slightly better ability to predict disease course, diagnosis, and disability and a good correlation with MRI findings [25, 26].

## 3. Structural MRI and Multiple Sclerosis

MRI is considered a valuable tool to evaluate the extent of brain damage in MS patients. Many efforts have been made to develop new MRI techniques able to detect not only WM matter lesions but also the extent of normal appearing white matter (NAWM) and gray matter (GM) [27]. The role of MRI is crucial in the diagnosis of MS and CIS, being MRI formally included in the diagnostic criteria [28]. Conventional MRI techniques have been extensively used in clinical practice, being fundamental in the assessment of disease activity and in the exclusion of alternative neurological conditions. T2-weighted and gadolinium-enhanced T1-weighted sequences are highly sensitive in detecting MS lesions, providing a quantitative assessment of inflammatory activity and lesion load [29]. The T2-hyperintense lesions represent various degrees of inflammation, demyelination, gliosis, edema, and axonal damage. They affect periventricular WM region, corpus callosum, the juxtacortical region, the infratentorial region, and spinal cord [30]. The use of intravenous gadolinium agent shows the disruption of the blood brain barrier and acute inflammatory lesions, appearing as bright areas on T1-weighted scans [31]. Fluid-attenuated inversion recovery (FLAIR) sequences, suppressing the signal from cerebrospinal fluid (CSF), are more sensitive than T2-weighted images in detecting periventricular and juxtacortical lesions, being less accurate in detecting posterior fossa lesions and spinal cord lesions, due to flow related artifacts [27]. A subset of T2 and FLAIR lesions may appear hypointense on T1-weighted sequences. These lesions are commonly called “black holes” (BH) and may range from mild hypointensity to severe hypointensity, similar to CSF signal. Based on longitudinal changes, BH originating from Gd-enhancing lesions may be classified as acute BH or persistent BH. The acute BH disappear in 6–12 months, reflecting edema and demyelination with subsequent recovery. Persistent BH reflect irreversible demyelination and axonal loss [32]. Brain atrophy, reflecting irreversible tissue loss, is another parameter of disease progression, usually quantified on T1-weighted images. Several methods are available for the measurement of global and regional brain volume. Normalized volumes are more adequate for cross-sectional studies, whereas absolute volumes measures are optimal for longitudinal measurement [33]. Volume loss occurs in both WM and GM and tends to correlate better with disability and CI than other MRI measures. In particular GM atrophy seems to be a better marker of disease progression compared to whole brain or WM fraction [11]. Although conventional MRI techniques are crucial in the MS diagnostic workup, their accuracy in evaluating and predicting disease progression is less relevant [34]. The introduction of quantitative MRI techniques improved our understanding of the mechanism of tissue damage, shedding light on the pathogenesis of the disease. Standard MRI techniques are not able to detect cortical lesions (CLs), so more advanced techniques have been developed. A multislab three-dimensional (3D) double inversion recovery (DIR) was applied to MS population with improvement of the detection of cortical damage [35]. Later on a new single slab, isotropic version of 3D-DIR was developed, in order to reduce the acquisition time and flow artifact [36]. In the last years quantitative measures have been developed in order to better quantify the burden of pathological changes occurring in MS patients. Magnetization transfer (MT), a measure based on the exchange of magnetization between protons bound to the brain tissue matrix and the surrounding “free” water, provides a quantification of subtle tissue damage, preceding by several months the appearance of lesions. MT ratio reflects demyelination and axonal loss. Decreased MT ratio in lesions and normal appearing (NA) WM/GM shows a good correlation with clinical disability [37]. Diffuse tensor imaging (DTI) MRI measures the random diffusional motion of water molecules, through the calculation of fractional anisotropy (FA) and mean diffusivity (MD) [38]. DTI is sensitive in detecting diffuse tissue damage in NAWM and NAGM. Furthermore DTI provides the basis for tractography, useful to assess the integrity of corticospinal tract and corpus callosum and the connectivity between regions [38, 39]. Proton MR spectroscopy (H-MR spectroscopy) is a quantitative MR technique able to provide chemical-pathological characterization of visible lesion and normal appearing brain tissue [40]. Various central nervous system (CNS) metabolites have been used as marker of pathological process in MS brain. In particular decreased levels of N-acetyl aspartate are considered a marker of neuroaxonal loss, while increased choline and myoinositol levels are marker of demyelination/inflammation and gliosis, respectively [41]. Other quantitative methods have been developed to better quantify iron deposition such as T2 relaxation time (RT) and susceptibility-weighted imaging techniques (SWI) [42, 43], although their utility in assessing brain damage needs to be fully investigated. In recent years imaging at an ultrahigh field (>3.0 T) provided great advantage in image contrast and resolution. In particular SWI sequences and in vivo MRS may benefit from imaging at an ultrahigh field providing new insights into the understanding of MS pathology [44, 45].

## 4. Gray Matter and Cognitive Impairment

In the last years, the classical view of MS as a WM pathology has been overcome by MRI techniques, able to detect the involvement of GM in the development of the disease. Nonconventional MRI technique showed that GM involvement can precede WM damage and is associated with physical disability and CI [46–48]. These findings were also confirmed by postmortem studies, showing that WM changes are accompanied by GM demyelination [49]. Demyelination can be found in other GM areas such as the thalamus, basal ganglia, hypothalamus, hippocampus, cerebellum, and spinal cord [50, 51]. Later, in vivo atrophy studies showed that GM changes could be detected in the early stage of the disease [13]. Interestingly, the burden of GM demyelination tends to become more prominent with the accrual of disability in the progressive stage of the disease [11, 52]. MRI has been widely used as a surrogate marker to differentiate patients with CI in MS. Several studies have shown a modest correlation between WM lesion load and cognitive performance, suggesting the involvement of other structures [8]. Studies about whole brain atrophy have shown a modest association with CI, whereas regional atrophy studies have demonstrated a better correlation with cognitive performance [9, 53, 54].

### 4.1. Cortical Lesions and Cognitive Impairment

Postmortem studies of MS brains have showed that demyelination can occur in every region of the CNS, including WM, cerebral cortex, and deep gray matter [55]. In recent years the use of immunohistochemistry for myelin protein demonstrated the presence of myelin in cortex of normal subjects and the extensive cortical demyelination in MS brains [56]. CLs have been described in pediatric MS patients, in CIS patients [57], in radiological isolated syndrome (RIS) [58], and early MS [59] suggesting GM damage can be detected in the very early stage of the disease. Based on these findings the detection of CLs in the MRI diagnostic criteria has been proposed [60]. Peterson et al. [61] proposed a classification of CLs based on histopathological findings. Type I lesions are close to subcortical WM lesions (juxtacortical lesions); type II are confined to the cortex, often perivascular (intracortical lesion); type III lesions extend from the pial surface to the cortical layers (subpial lesions). A combined histopathologic MRI study confirmed that not all CLs can be detected with DIR, in particular the type III subpial lesions. The implementation of high field MRI demonstrated an increased sensitivity in detecting subpial lesions [62]. The correlation between CLs load and CI has been investigated with differing results. A study combining two different MRI techniques, such as DIR and T1-weighted phase-sensitive inversion recovery (PSIR), showed that both intracortical lesion and mixed lesions play a more significant role than juxtacortical lesions and measures of atrophy in CI [63]. A longitudinal study showed in a small group of MS patients (13 subjects) that CLs tend to increase over time and were associated with a cognitive decline. In particular the authors observed a significant correlation between hippocampal lesion load and the location learning test score (LLT), investigating visuospatial memory [64]. Mike et al. [65] found a similar correlation between CLs load, WM volume damage, and SDMT. They also found CLs were a good predictor of verbal learning and memory assessed by California verbal learning test (CVLT-II). Calabrese et al. [12] demonstrated that CL, GM damage volume, and age are good predictors of CI, showing also a better correlation of CI with CL than WM damage in a group of MS patients. Papadopoulou et al. [66] did not confirm these findings, demonstrating that WM lesion volume plays a major role in the development of CI compared to CLs.

### 4.2. Gray Matter Atrophy and Cognitive Impairment

#### 4.2.1. Cortical Atrophy

GM atrophy represents a marker of degeneration in MS patients. It occurs early in the disease course and tends to progress over time to a greater degree than whole brain and WM atrophy [46]. High-resolution MRI and automated segmentation techniques have been used to quantify GM tissue volume and to improve our understanding of the pathogenic mechanism responsible for GM damage. The assessment of GM atrophy and its topographic distribution may help to correlate cognitive domain to a specific brain region [67]. The pathogenic mechanism responsible for cortical atrophy needs to be fully elucidated. Nevertheless, it is believed that not only demyelination is responsible for GM atrophy, but also axonal transection, neuronal, glial, and synaptic loss can be found in cortical GM lesions and could be responsible for atrophy and cortical thinning in MS [68]. Several authors investigated the role of GM and its association with CI. In a group of RRMS patients, Amato et al. [69] found that cortical atrophy was correlated with a poor performance on NPS testing. In particular neocortical atrophy was associated with impairment in verbal memory, verbal fluency, and attention. After a followup of 2.5 years the authors found higher changes in cortical volume in cognitively impaired patients compared to the cognitively preserved ones. Benedict et al. [70] have shown the correlation between neocortical volume and many neuropsychological measures (verbal and visuospatial, memory, processing speed, and working memory). Also they demonstrated the third ventricle width, reflecting thalamic atrophy, could be considered a good predictor of CI. The association of cortical atrophy with slower speed and memory impairment, controlling for III ventricle width, demonstrated that both central and cortical atrophy were predictor of CI in MS [71]. Gioia et al. [72] showed in a group of RRMS patients a significant difference in GM volume between cognitively impaired patients and cognitively preserved while no differences were found in WM volume. Sanfilipo et al. [53] investigated the different role of WM and GM in cognition. They suggested that WM loss is associated with impaired processing speed and working memory, while GM is more closely involved in impaired verbal memory. A study by Calabrese et al. [73] showed two different patterns of cortical thinning between cognitively impaired patients and cognitively preserved. They found a widespread atrophy in cognitively impaired patients while a frontotemporal thinning in cognitively preserved patients, suggesting that probably the involvement of GM in the pathology of CI starts in these regions. The involvement of certain area of gray matter and the association with specific domain have been investigated. In a group of RRMS and SPMS patients it has been shown that temporal lobe atrophy is associated with a poor outcome in memory performance while whole brain or central atrophy is more related to processing speed performance [74]. Another study about regional atrophy showed a correlation between hippocampal atrophy and a poor performance in memory-coding test [75]. Regional volumetric analysis has been used to better characterize the topographical distribution of GM atrophy [76]. Voxel-based morphometry (VBM) is a technique able to identify difference in the local composition of brain tissue, comparing voxel by voxel features between subjects group and correlating voxel features with relevant subject variables [77]. The use of this technique has allowed investigating the relationship between CI and GM regional atrophy. Morgen et al. demonstrated a correlation between PASAT score and global GM volume. In particular they found a correlation with regional atrophy in a number of cortical areas, such as prefrontal cortex, precentral gyrus, superior parietal cortex, and right cerebellum [78]. These findings were confirmed by a recent study investigating cognition in MS patients, using VBM and tract-based spatial statistics (TBSS) to assess regional GM and WM damage, respectively. They found a correlation between GM volume and PASAT in orbito-frontal cortex, while TBSS showed significant correlations between DTI metrics and PASAT scores in many WM tracts including corpus callosum, internal capsule, posterior thalamic radiations, and cerebral peduncles [79]. In another study Mesaros et al. [80] did not find any difference in regional gray matter atrophy between cognitively impaired benign MS patients and SPMS patients. Riccitelli et al. showed a different pattern of GM atrophy between cognitively impaired RRMS and SPMS patients. They found a prominent involvement of deep GM region in RRMS whereas a major involvement of cortical area in SPMS patients [81].

#### 4.2.2. Subcortical Atrophy

During the course of the disease, cortical and subcortical demyelination has been observed in GM structures, including thalamus, caudate, putamen, globus pallidus, and other structures of the basal ganglia [82]. It has been postulated that deep gray matter atrophy is not the result of a direct damage from MS pathology, but it is the consequence of the disconnection via axonal transection within WM damage [83, 84]. Between the structures of the deep GM, the thalamus has shown the strongest correlation with CI. The loss of volume in the thalamus has been demonstrated in CIS patients [85] and pediatric MS patients [86], representing one of the earlier markers of subcortical GM pathology. Atrophy of the thalamus has also been described in different stages of the disease. Houtchens et al. [87] found a 16.8% reduction of thalamus volume in MS patients compared to control. Moreover, thalamus was the stronger predictor of memory and processing speed performance. Another study of an independent group confirmed these data and found that putamen atrophy may also play a role in the development of CI [88]. Using a high field strength 3T MRI, a significant association between memory, deep gray matter structures, and cortical thinning of the frontal and temporal gyrus was demonstrated in a group of MS patients [89]. A recent study investigated the role of sex in cognition and subcortical gray matter in a group of early relapsing MS patients. They found all cognitive domains except visuospatial memory were affected in men; none were significantly affected in women. Deep GM volume was more affected in men compared to women with bilateral hippocampus, amygdala, and right nucleus accumbens in men and right hippocampus and nucleus accumbens, bilateral amygdala, and putamen in women, showing no atrophy compared to controls. These findings, again, underline the involvement of deep GM damage in CI and the relevance of a sex specific atrophy mechanism in MS [90].

## 5. GM Diffuse Damage and Quantitative MRI Technique

Over recent years many studies started measuring abnormalities in NAGM by using various quantitative MRI techniques such as MTR, T1 relaxation time measurements, DTI, and MRS [67]. The recent use of high-field scanners has improved our ability to detect and quantify such abnormalities. Changes in MT ratio correlate with overall cognitive performance better than lesions or atrophy. In particular a correlation has been shown between PASAT score and left and right Brodmann area, right superior longitudinal fasciculus, and splenium in a group of CIS patients [91]. Another study investigating the extent of CI in CIS patients showed cortical MT ratio was the only MRI parameter associated with impaired mental processing speed, suggesting cortical MT-ratio changes may be considered as a biomarker of tissue damage in the very early stage of the disease [92]. A strong association between GM MTR and a worse overall cognitive performance was demonstrated in progressive patients [93]. Another study using a voxel-based method confirmed these findings in primary progressive MS, showing significant correlations between decrease of MTR value in specific cortical regions and PASAT performance [94]. A recent 13-year follow-up study showed GM MTR was the only MRI predictor of global CI, supporting the notion that GM plays a major role in the long-term development of CI [95]. GM diffuse damage can also be investigated with DTI. A DTI study in a group of mildly disabling RRMS patients has demonstrated a moderate correlation between GM MD and the degree of CI, in the absence of correlation with physical disability [96]. Using a voxel-based approach, Ceccarelli et al. found GM DTI abnormalities in brain area (thalami, right insula) associated with cognition in a group of PPMS, explaining in part the discrepancy between the low brain lesion load and the severe clinical status of progressive patients [97]. A recent study investigated the correlation between fatigue, CI, and damage in the anterior thalamic tract and corpus callosum, measured by DTI, in a group of benign MS patients. The authors showed that fatigue was associated with increased MD of the anterior thalamic tracts while impaired executive functions and verbal learning were associated with decreased FA in corpus callosum. Impairment of processing speed and attention was associated with T2 lesion volume in the anterior thalamic tract [98]. Several studies have found metabolite abnormalities in the cortical and subcortical gray matter in MS patients [67]. A significant reduction of N-acetylaspartate (NAA), a marker of neuronal damage, measured in the frontal cingulate gyrus, has been found to correlate with global memory functions in a group of early MS patients [99]. In another study Chard et al. [100] found significantly reduced NAA, choline (Cho), and glutamate-glutamine (Glx) in cortical GM. They also observed a significant correlation between MS functional composite score and the metabolites concentrations. In particular PASAT score showed a significant correlation with cortical GM Glx. T1- and T2-based measures allowed the quantification of GM microscopic damage, usually not detectable with conventional MRI technique [101]. T2 hypointensity in the GM, usually seen in MS patients, has been related to iron deposition. Paramagnetic substance, like iron, reduces T2 relaxation time, resulting in hypointensity in T2 images. T2 hypointensity has been described in the red nucleus, thalamus, dentate nucleus, lentiform nucleus, caudate, and rolandic cortex. Brass et al. showed that T2 hypointensity of the globus pallidus was most closely associated with overall cognitive performance [102] (see Table 1 for MRI outcomes and cognitive functions investigated).

## 6. Conclusions

Following Charcot neuropathological studies, the involvement of GM in the pathogenic mechanism of MS has been described [103]. Conventional MRI techniques are not able to detect cortical lesion, cortical and deep GM atrophy. In the last years the use of new MRI techniques has improved our understanding of the mechanism responsible for CI in MS patients. Many studies have shown a correlation between cortical lesions, cortical and deep GM atrophy, and cognition although a clear localization of a cognitive domain on a specific brain region has not been clearly demonstrated. Although in the last years many efforts have been made to better clarify the correlation between GM damage and cognition in MS, many questions are still unanswered. Large MRI longitudinal studies are needed to better understand the development of CI and to evaluate the temporal evolution of associated tissue damage and the role and the reciprocal influence of WM and GM structures. New MRI techniques and postprocessing methods are confined in research field and have not yet been fully implemented in clinical routine. Based on this the use of standardized acquisition of MRI sequences between scanner manufacturers and different centers is crucial and should be considered in the future. The understanding of cognitive pathway and the use of MRI as a surrogate marker of CI are needed in order to better investigate the evolution of this disabling symptom and preserve cognitive function. The definition of a valid and sensitive MRI biomarker is crucial to clarify the pathogenesis of CI and to monitor the neuroprotective effects of novel drugs.

## Figures and Tables

**Table 1:** Studies investigating the role of MRI measures and cognition in MS.

Author (year)	MRI measures	Cognitive findings
Morgen et al. (2006) [78]	Brain atrophy	Executive functions
Amato et al. (2007) [69]	Percentage of brain volume changes, NVC, normalized deep GM volume change, and LL	Overall CI
Calabrese et al. (2009) [12]	CLs, WM lesion volume, CELs, NBV, and NCV	Overall CI
Nelson et al. (2011) [63]	CLs	Overall CI
Mike et al. (2011) [65]	WM lesion volume, CL number, and CL volume	Overall CI
Khalil et al. (2011) [92]	Cortical MT ratio	Information processing speed
Batista et al. (2012) [88]	Neocortical, deep GM volume	Information processing speed
Sbardella et al. (2013) [79]	Regional GM and WM atrophy	Executive functions

GM: gray matter, NCV: normalized neocortical GM volume, LL: lesion load, CI: cognitive impairment, CLs: cortical lesions, WM: white matter, CELs: contrast enhancing lesions, NBV: normalized brain volume.
